# Grass-roots entrepreneurship complements traditional top-down innovation in lung and breast cancer

**DOI:** 10.1038/s41746-021-00545-x

**Published:** 2022-01-21

**Authors:** Khalil B. Ramadi, Rhea Mehta, David He, Sichen Chao, Zen Chu, Rifat Atun, Freddy T. Nguyen

**Affiliations:** 1 Hacking Medicine, Massachusetts Institute of Technology, Cambridge, MA USA; 2 School of Engineering, Massachusetts Institute of Technology, Cambridge, MA USA; 3 Harvard T.H. Chan School of Public Health, Harvard University, Boston, MA USA; 4 Division of Engineering, New York University Abu Dhabi, Abu Dhabi, UAE; 5 Tandon School of Engineering, New York University, New York, NY USA; 6 Department of Economics, Wellesley College, Wellesley, MA USA; 7 Sloan School of Management, Massachusetts Institute of Technology, Cambridge, MA USA; 8 Innovation Initiative, Massachusetts Institute of Technology, Cambridge, MA USA; 9 Institute for Medical Engineering and Science, Massachusetts Institute of Technology, Cambridge, MA USA

**Keywords:** Cancer, Health care economics, Health policy

## Abstract

The majority of biomedical research is funded by public, governmental, and philanthropic grants. These initiatives often shape the avenues and scope of research across disease areas. However, the prioritization of disease-specific funding is not always reflective of the health and social burden of each disease. We identify a prioritization disparity between lung and breast cancers, whereby lung cancer contributes to a substantially higher socioeconomic cost on society yet receives significantly less funding than breast cancer. Using search engine results and natural language processing (NLP) of Twitter tweets, we show that this disparity correlates with enhanced public awareness and positive sentiment for breast cancer. Interestingly, disease-specific venture activity does not correlate with funding or public opinion. We use outcomes from recent early-stage innovation events focused on lung cancer to highlight the complementary mechanism by which bottom-up “grass-roots” initiatives can identify and tackle under-prioritized conditions.

## Introduction

Biomedical research enhances our understanding of disease and helps to develop more effective methods to decrease disease incidence, morbidity, and mortality. Public funding mechanisms have historically provided the majority of early-stage biomedical research funding worldwide^1^. In the United States (US), the National Institute of Health (NIH) is the single largest funder of biomedical research, with ~80% of its budget allocated to extramural grants^2^. Strategic grant offerings by public, non-profit, and private entities can have a substantial impact in defining and kick-starting the development of innovations in specific research areas. For example, the Brain Research through Advancing Innovative Neurotechnologies (BRAIN) initiative launched in 2013 with a focus on neuroscience research, led to a major intensification of efforts in neuroimaging, computational neuroscience, and neurodegenerative disease^3^.

Prioritization of funding allocations across diseases is a complex process^4–6^. From economic allocative efficiency and societal perspectives, conditions contributing to the most significant disease burden should be prioritized. However, public awareness and attitudes can play major roles in shaping prioritization decisions, as can private interests and incentives^7,8^. Such awareness and attitudes can fluctuate over time.

Factors considered in prioritizing research include “push” metrics that may make certain diseases more attractive to work on (such as societal impact and disease burden, amount of funding needed to make meaningful progress, understanding of the nature of the disease (e.g., etiology)), and “pull” metrics that incentivize successful innovation based on outcomes (such as commercial interests, public interest, sentiment, and support). In general, push metrics spur the onset of innovation while pull metrics reward outcomes. Prioritization through such “push” and “pull” between economics, public interest, and specific incentives of individual groups can leave gaps in funding areas that are not deemed to be potentially impactful.

In this study, we focused on funding disparities between cancer subtypes in the US. Cancer is the second leading cause of death in the US^9,10^. Among the more than 30 subtypes of cancer, breast and lung cancers are the two most common cancers worldwide and in the US^11^. Breast and lung cancers are case examples of two conditions with a major disparity between funding and disease burden. We characterize the extent of this disparity in diagnosis, therapy, and clinical trials, and compare public sentiment and venture activity in each field. We introduce bottom-up innovation programs as complements to help address this disparity without necessarily redirecting or restructuring traditional mechanisms. Alternative innovation models can complement traditional top-down funding mechanisms (such as grants and “Request for Proposals” (RFPs)), and in doing so help efficiently redistribute innovation efforts across diseases. Outputs of early-stage innovation events are able to incorporate novel technologies as tools to address pain points in lung cancer screening and diagnosis that remain unaddressed through traditionally funded research programs.

## Results

### A tale of two cancers: prevalence, mortality, burden of disease, and funding allocations

Breast and lung cancers are the two most common cancers worldwide^11^. We found that lung cancer is the second most common cancer in men and women in the US with, on average, 62 in 100,000 people diagnosed each year since 1980^12^. By comparison, on average 70 in 100,000 people are diagnosed with breast cancer each year, and is the most common cancer in women in the US (Supplementary Fig. 1a)^12^.

**Fig. 1:**
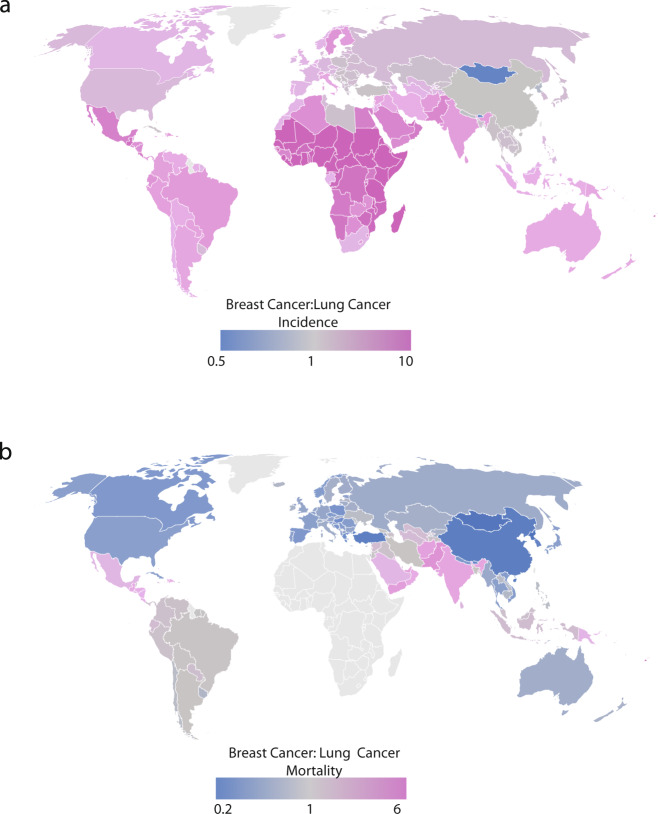
Global burden of breast and lung cancers. **a** Worldwide incidence and **b** mortality for breast and lung cancer in 2018. Data are shown as the ratio of breast cancer to lung cancer. Values higher than 1 (pink) or less than 1 (blue), respectively reflect greater breast cancer, or lung cancer, incidence/mortality. Mortality data for African countries are shown in Supplementary Fig. 2 for clarity due to high breast to lung ratios for those countries. Maps generated using Google GeoChart API under Creative Commons Attribution 4.0 License.

The worldwide incidence of breast cancer is generally higher than that of lung cancer (Fig. 1a). Despite this, however, lung cancer has a much higher mortality rate worldwide (Fig. 1b and Supplementary Fig. 2). In the US, lung cancer has an average yearly mortality rate three times that of breast cancer (53/100,000 vs. 16/100,000) (Fig. 1b and Supplementary Fig. 1b)^12^. In 2017, deaths due to lung cancer in the US exceeded those of colon, breast, and prostate cancer combined^13^.

Metrics to quantify the socioeconomic cost of the disease include years of life lost (YLL), which is the total sum of years of life taken away from all patients with the disease, and disability-adjusted life years (DALYs), which aggregates the number of years lost due to ill-health or early death. We found that the YLL globally due to lung cancer is 60% more than breast cancer (1,104,000 vs. 680,200 YLL), while lung cancer DALYs are almost triple that of breast cancer (13.26 vs 4.86 million years)^14^. Higher DALYs signals ineffective diagnostic and treatment methods^15^. This disparity exists notwithstanding a substantial recent downward trend in the incidence of lung cancer^16^.

**Fig. 2:**
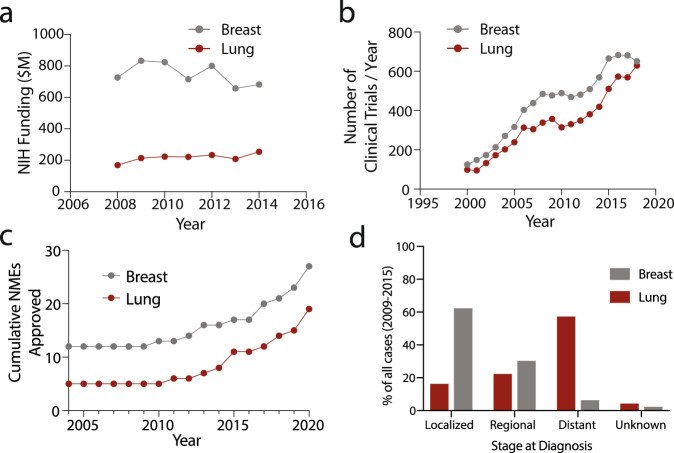
Research and development activity in breast and lung cancers. **a** US National Institutes of Health (NIH) funding for breast and lung cancers. **b** Total number of active clinical trials per year for breast and lung cancers. **c** Cumulative new molecular entities (NMEs) approved by the FDA from 2004 to 2020. Cumulative number calculated from NME approvals starting 1985. **d** Percentage of cases based on the stage at diagnosis of both breast and lung cancer (2009–2015).

Given this disparity in disease burden, we investigated NIH funding data from 2008 to 2014. We found that breast cancer has received 3.5 times more funding that lung cancer (Fig. 2a). There are also 23% fewer clinical trials globally for lung cancer than for breast cancer over 2000–2018 (Fig. 2b), as well as fewer approved pharmacotherapies by the US Food and Drug Administration (FDA) (Fig. 2c). While the burden of disease can play a role in setting research priorities, it is clearly just one of many factors that are considered in prioritization decisions.

Stage of disease at diagnosis could influence mortality. We found that the majority (62%) of breast cancer cases were diagnosed when the cancer was still localized (Fig. 2d). In contrast, 58% of lung cancer cases were diagnosed after metastasis, leading to substantially more difficult-to-treat cases and likely resulting in higher mortality. This suggests that the high social burden of lung cancer may be due to a dearth of early-stage diagnoses. This is also consistent with the lack of a means for direct physical evaluation of lung tissue. There is no equivalent to breast exams that can be self-administered or conducted by a medical provider.

### Public awareness and sentiment of breast vs. lung cancer

**Fig. 3:**
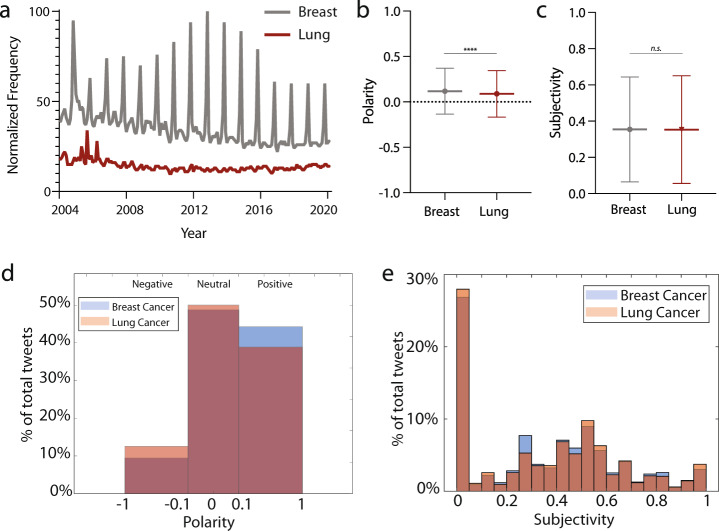
Social media trends and sentiment. **a** Normalized search engine search frequency for “Breast Cancer” and “Lung Cancer” in the US. **b** Average polarity and **c** subjectivity of tweets for breast and lung cancer. Error bars signify Standard Deviation. **d** Percentage of breast and lung cancer tweets with negative (−1 to −0.1), neutral (−0.1 to 0.1), and positive (0.1 to 1) sentiment. **e** Percentage of breast and lung cancer tweets with different levels of subjectivity (0 to 1 in increments of 0.05). ****P < 0.0001; n.s. not significant, unpaired *t* test with Welch’s correction.

Early-stage diagnosis of breast cancer has been shown to be improved due to robust social awareness campaigns and widespread availability of screening^17,18^. As such, we examined social awareness and public sentiment of breast and lung cancers as possible correlates with public funding levels. We analyzed Google trends data as a proxy for public awareness^19^. Our analysis revealed that searches for “breast cancer” were twice as common as those for “lung cancer” over the past 15 years. Moreover, there was a twofold increase in searches for “breast cancer” in October of every year, reflecting the month’s status as Breast Cancer Awareness Month (Fig. 3a). Breast cancer cyclical interest peaks in October far exceeds public interest in other diseases on their respective awareness months, including for lung cancer (Supplementary Fig. 3). Such high levels of social awareness play an important role in disseminating information about symptoms, encouraging screening, and educating on lifestyle factors to minimize risk^20^. One possible factor contributing to more robust public awareness campaigns for breast, as opposed to lung, cancer may be the younger age at diagnosis (62 years for breast vs 71 years for lung)^21^.

Public opinion and stigma for cancer can also decrease an individual’s likelihood to seek screening for early symptoms^22^. We sought to compare public sentiment for breast and lung cancers. We scraped and applied sentiment analysis to 28,126 tweets during 2019 that included “breast cancer” and “lung cancer” as keywords, maintaining approximately equal numbers of tweets for each to avoid bias. We assumed that for any tweet that mentions “breast cancer” or “lung cancer”, the sentiment value of the tweet was directed at that cancer type. Similar to previous NLP studies^23–25^, we then infer relative opinion towards breast and lung cancers by comparing the aggregate sentiment values of tweets mentioning each. Breast cancer tweets had more positive sentiment on average than lung cancer tweets (polarity 0.118 vs. 0.088 (*P* < 0.0001); Fig. 3b). There was no difference in subjectivity between breast and lung cancer tweets (subjectivity 0.354 vs 0.353 (*P* = 0.7416); Fig. 3c). We also found that negative sentiment was contained in substantially more lung cancer (1735/13995 total; 12.4%) than breast cancer tweets (1303/14131 total; 9.2%). Breast cancer tweets also more often carried positive sentiment (5775/14131, 40.8%) compared with lung cancer (4995/13995; 35.7%). (Fig. 3d). No differences in subjectivity were found in tweets with either condition (Fig. 3e).

### Venture activity and startup formation in breast vs. lung cancer

**Fig. 4:**
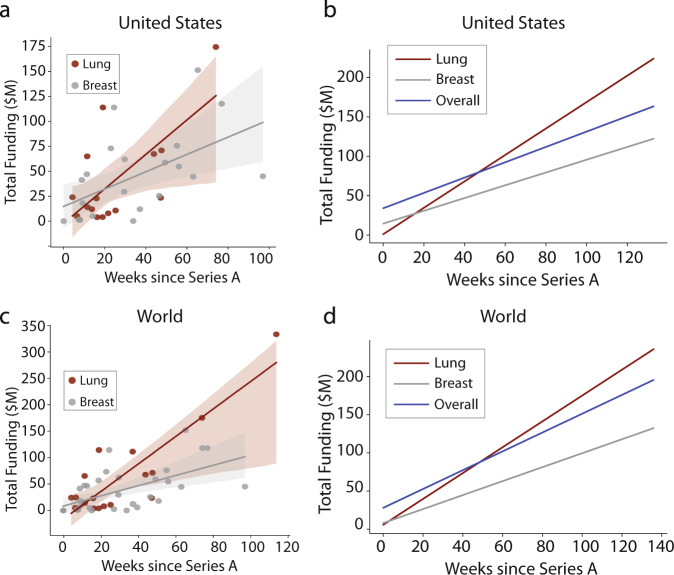
Funding raised by cancer ventures. **a** Cumulative venture funding raised over time for lung and breast cancer companies in the United States, and **b** lung, breast, and overall cancer companies. **c** Cumulative venture funding raised over time for lung and breast cancer companies worldwide, and **d** lung, breast, and overall cancer companies. Lines reflect linear regressions.

The translatability of biomedical research can be assessed by examining venture activity in any given field or disease area. We compared the growth trajectories of early-stage companies (defined as private companies that have not offered an initial public offering (IPO)) developing diagnostics or therapeutics for breast and lung cancer. There were no significant differences in the number, or growth trajectories, of companies in either field (Fig. 4a, c). In fact, early-stage companies working on lung cancer-related products and solutions experienced slightly accelerated rates of growth. This trend was also evident when compared to all early-stage companies with cancer-related products and solutions worldwide and in the US (Fig. 4b, d). These findings suggest that the impact of top-down funding does not necessarily translate to a greater number of ventures or accelerate venture funding in the field. On the contrary, venture activity reflects unmet market needs and compensates by incentivizing the private sector to fill gaps left by public funding.

### The role of bottom-up innovation activity

**Fig. 5:**
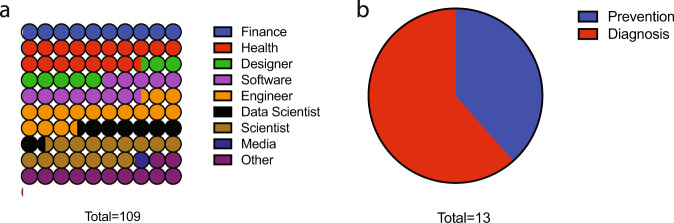
MIT HM 2018 Health hackathon data. **a** Participant background and **b** solution approach by the team at an early-stage innovation event focused on lung cancer.

Given the relative paucity of funding and evidenced market need for lung cancer technologies, we next examined the impact of an early-stage innovation event in bringing together diverse stakeholders to develop new innovations for lung cancer. A healthcare hackathon organized by the Massachusetts Institute of Technology (MIT) Hacking Medicine (MIT HM, Box 1 and “Methods”) was run in November 2018, focused on addressing outstanding problems in lung cancer. Stakeholders included patients, providers, students, and professionals with a wide array of backgrounds, including health, finance, engineering, and design (Fig. 5a).

Over the 3-day event, participants formed multidisciplinary teams and pitched their solutions to judges. Teams were allowed to tackle any problem related to lung cancer. Over 50% of teams developed solutions focused on early diagnosis and detection, identifying the major need in lung cancer (Fig. 5b and Supplementary Table 1). Four months after the event, we found that 29% of teams continued working on their technologies. Teams leveraged diverse skillsets to utilize technologies including data-driven artificial intelligence (AI) and machine learning (ML) in addressing problems of diagnosis and detection (see Box 1 and Supplementary Table 1 for examples). Challenges reported by teams who did not continue in advancing their ideas centered on fundraising, clinical collaborators, and time commitment.

Box 1. Hackathons and grass-roots innovation Early-stage innovation events can be tailored to specific diseases or objectives^40–45^. These events assemble diverse stakeholders and nurture teams with dedicated time and expertise to brainstorm and develop solutions to problems. A subset of such innovation events, we describe hackathons as a bottom-up, or “grass-roots”, innovation approach. Hackathons encourage the fresh perspective of non-experts as crucial to developing new innovations, and can democratize innovation in the absence of robust top-down mechanisms^46^. Hackathons serve as a bridge between academia, industry, and healthcare realms^47^, forming a nucleus for private-public partnerships (PPPs) to gather academic research, public resources, and private funds, and translational expertise towards the development of new health innovations. PPPs are a powerful tool to address gaps in healthcare systems globally, including access to care and the development of new therapies^48^.

Hackathons can complement traditional funding mechanisms by energizing and empowering a diversity of stakeholders. The intentional mixing of disciplines can also have a rejuvenating effect on a field. One company that has arisen from the MIT Hacking Medicine 2018 lung cancer hackathon event, CanAIry, is developing artificial intelligence algorithms to enable early detection of lung cancer by a smartphone app-based purely on a cough (https://www.canairy.ai). If successful, this could have a profound effect on lung cancer screening and diagnostics. Such innovations can further incentivize other outsiders to the field to also become involved. Other teams at the 2018 event focused on nonmedical factors, including lifestyle improvement, diet, exercise, and smoking cessation. These incorporated expertise of psychology and marketing for behavior modulation. In hackathons, most teams are focused on a specific context where their solution can be quickly deployed and tested. This contrasts with most top-down grants that are predicated on being applicable to a broad audience. While traditional funding mechanisms focus on diseases with greater public awareness, bottom-up innovation shifts the focus on under-prioritized diseases and gaps in the traditional top-down pipeline.

## Discussion

Diseases with a higher social burden do not necessarily receive a greater proportion of research funding. We identified a disparity between disease burden and funding levels for breast and lung cancers. The effect of funding discrepancies can propagate downstream, limiting the number of clinical trials for each disease. Similar burden-funding disparities have been previously characterized. What has been less studied is why these disparities arise. Using social media and aggregated internet search histories to quantify public sentiment and awareness around each condition, we found that lung cancer has significantly less public awareness and is associated with a more negative opinion when compared to breast cancer. These correlations give some indication as to how public funding correlates with public sentiment. This, in turn, is influenced not only by the prevalence of the disease but also by informational campaigns such as awareness months, as well as potential media bias and stigma.

Interestingly, our findings suggest that venture activity for each disease is somewhat immune to this bias. Growth trajectories of ventures in lung cancer are increased when compared to breast cancer ventures. This accelerated growth is also greater than the average of companies working on any other subtype of cancer. Venture growth seems to more objectively reflect market need, suggesting that developing new translatable innovations could directly stimulate investment and help develop products and solutions to improve disease management. One way of achieving this is through early-stage innovation events. Such events can energize and empower non-expert individuals to contribute to solutions for diseases that may be under-addressed (see Box 1). In the case of lung cancer, such events can focus on developing new screening tools to enable diagnoses at earlier, pre-symptomatic stages of the disease.

Social media and automated NLP methods have been used in various applications for better real-time understanding of public or grassroots sentiments. For example, a digital diplomacy index was developed targeting official Twitter accounts of national governments and their leaders to provide a window into the world of digital diplomacy^26^. Sentiment analysis has also been applied extensively in healthcare settings, to gauge public perception of new treatments, vaccines, and policies^27–31^. One recent report used social media posts to study and track real-time temporal and geolocation of opioid-related social media posts as a proxy to monitor the larger opioid epidemic^32^. Our approach combines publicly available search engine histories and social media posts to estimate the public sentiment of disease.

Our study has a number of limitations. In quantifying research funding levels, we have limited our scope to the US NIH. There exist other governmental and nongovernmental agencies that fund research, which we have not included in our analyses. Our study incorporates a number of new methods leveraging the internet and social media to discern public opinion. We illustrate how these tools enable access to large amounts of text data, which can then be analyzed using automated NLP algorithms. Despite attempts to validate algorithm performance on a subset of data, it remains difficult to do so in the absence of an objective metric of “sentiment”. Our assumption that computed sentiment of tweets is correlated with public opinion is consistent with prior sentiment analysis studies^25^. For example, Matalon et al. used tweet sentiment using a standard natural language toolkit (NLTK) as a proxy for an individual opinion in political communications^23^. O’Connor et al. inferred public political opinion from subjectivity and polarity of tweets analyzed using sentiment analysis and showed it to be as reliable as traditional polling methods^33^. However, as with these previous studies, equating sentiment with opinion may not always be appropriate. Finally, our study does not examine the causality of relationships between funding and public sentiment. It is possible that top-down priorities elevate certain fields to be more featured in the media, which in turn leads to more public awareness.

We also used a variety of datasets in this study with distinct timespans: disease incidence and mortality (1980–2019), NIH data (2008–2014), clinical trials (2000–2019), FDA data (2004–2020), search engine results (2004–2020) and social media tweets (2019). In most of these ranges, trends do not differ significantly over time. For example, NIH funding was four times higher for breast as opposed to lung cancer in 2014, just as it was in 2008. “Breast cancer” has been twice as frequently searched on Google than “lung cancer” throughout the past decade. However, while lung cancer mortality remains more than double that of breast cancer, lung cancer incidence has decreased ~17% since 2010. Our analysis does not take such trends into account.

**Fig. 6:**
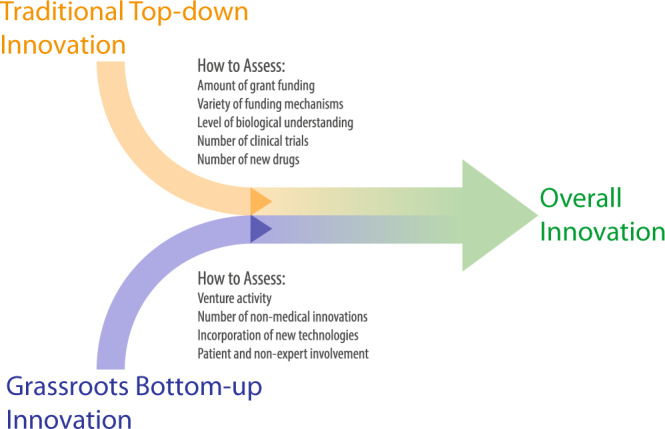
Combining top-down and bottom-up innovation. Complementary roles for traditional top-down and grassroots-driven innovation and and metrics to assess the activity of each.

Traditional funding mechanisms are important to establish a certain threshold of biological understanding of a disease. Once this threshold is reached, however, introducing outsiders to the obstacles faced in diagnosis and treatment through bottom-up innovation events can be a powerful method to find and fill gaps that remain, empowering patients and outsiders with a vested interest in the field. We introduce a framework pairing both tactics along with levers and metrics to, respectively, energize and analyze activity in each (Fig. 6). Top-down innovation is defined as traditional grant mechanisms where proposals are submitted in response to specific “Request for proposals” announcements. These are appropriate for research-intensive innovations to advance basic scientific understanding. The impact of this can be measured by comparing levels of funding and the number of RFPs with the number of clinical trials or new drugs.

Bottom-up innovation is crucial to sustain translational efforts beyond research laboratories. This is particularly the case for nonmedical interventions seen in digital health, for example, which do not follow the traditional translational pipelines that new therapies do. Bottom-up innovation is also an efficient way to enable non-experts (including patients and their families) to contribute to outstanding issues in the field. This can also serve to incorporate and translate technologies across fields that have been traditionally siloed (e.g., machine learning in medicine). The level of bottom-up innovation in a field can be assessed by looking at venture investments in nondrug interventions (Fig. 6).

The utility of using hackathons to promote bottom-up innovation can be varied and context-dependent. Hackathons excel at democratizing the innovation process across participants of different backgrounds and disciplines. These events, however, need to be one component of an overall ecosystem to generate and nurture innovations. Hackathons have been criticized for being too short to generate lasting progress for complex healthcare issues^34^. Indeed, teams from various hackathons have reported that lack of follow-up funding is a primary reason for the abandonment of their idea. Leveraging a hackathon to its full extent could entail pairing teams with local and regional incubators that may be able to further support.

Shifting funding levels across diseases can be challenging due to social and political factors. In contrast, bottom-up innovation can invigorate interest in a specific field without having to significantly increase research funding. Innovation initiatives can incorporate both private and public partners, leveraging the resources of each. Governments stand to gain by contributing to the improvement of health for citizens, while companies can license intellectual property generated and retained by participants, in order to scale and monetize innovations. Early-stage innovation events foster entrepreneurship, an important driver for economic growth and prosperity^35,36^. Specific conditions that are underinvested in could benefit from early-stage innovation events to generate interest and catalyze the development of ideas and solutions for that condition. Small teams of entrepreneurs can de-risk ideas more readily than can larger traditionally funded firms.

In this way, programs incentivizing grassroots innovation can yield significant returns to governmental agencies at a lower cost than research funding. This is exemplified by initiatives taken to tackle the COVID-19 pandemic^37,38^. Virtual hackathons focusing on COVID-19 were able to attract a significantly larger number of participants at a fraction of the cost of in-person events. These events can effectively crowdsource solutions across geographies and backgrounds. Combining bottom-up innovation with traditional top-down funding can leverage the complementary benefits of each to establish and maintain steady progress in the fight against major diseases such as cancer.

## Methods

### Data sources and analysis

We obtained data from multiple sources for this study. Worldwide data on incidence and mortality of breast and lung cancers was gathered from World Health Organization databases^39^. Lung cancer incidence and mortality are 2018 estimated age-standardized rates for all ages and both sexes. Breast cancer incidence and mortality are 2018 estimated age-standardized rates for females of all ages.

We used YLL or DALYs as metrics to quantify the burden of disease.

YLL was computed as: *Disease burden (YLL)* *=* *(average life expectancy* *–* *average age at death)* × *number of deaths*

Average life expectancy, age at death, and the number of deaths were obtained from US National Institutes of Health SEER program data^12^. The most recent NIH funding information per condition was reported for the 2008–2014 interval. DALY incorporates YLL and years of life impacted by the disease or disability (YLD). We used data from the Global Burden of Disease study^14^ to quantify DALY’s. Clinical trials data was obtained from ClinicalTrials.gov (https://clinicaltrials.gov). Social awareness metrics were gathered from Google Trends data showing the normalized frequency of searches for “breast cancer” and “lung cancer” from 2004 to 2019 (https://trends.google.com/trends). This is the maximum range of data available from Google. Search frequency is normalized to the maximum search frequency in each particular graph.

Public sentiment data were obtained from public tweets using a Twitter scraper algorithm (https://github.com/taspinar/twitterscraper). Tweets including the key phrase “Breast Cancer” and “Lung Cancer” from January 1, 2019 to December 31, 2019 were obtained (n_Breast_ = 14,131, n_Lung_ = 13,995 tweets). We used the standard Twitter Tweet API v1 and obtained the maximum number of tweets possible to scrape. We estimate that the 28,126 tweets retrieved are 5% of the total relevant tweets. A python library for natural language processing (NLP) of textual data (TextBlob) was utilized for sentiment analysis (https://textblob.readthedocs.io/en/dev).

Textblob was built upon the standard natural language toolkit (NLTK) and it contains two sentiment analysis implementations, PatternAnalyzer (based on the pattern library) and NaiveBayesAnalyzer (an NLTK classifier trained on a movie reviews corpus). We used an external module that was trained on alternative data. (Documentation can be found at https://buildmedia.readthedocs.org/media/pdf/textblob/latest/textblob.pdf).

We evaluate the polarity and subjectivity of the text associated with each tweet was evaluated. Polarity is on a scale from [−1, 1], with lower numbers being more negative and higher numbers as more positive. Subjectivity is on a scale from [0, 1], with lower numbers from more objective tweets and higher numbers from tweets with more subjectivity. Data was exported and plotted using custom MATLAB^®^ (version R2018a) scripts (https://www.mathworks.com/products/matlab.html).

We used the Crunchbase database to obtain activity on venture activity across all time (https://www.crunchbase.com). The first set of data was obtained by applying three filters: (1) headquarters location in the US, (2) at least two funding rounds, and (3) keywords in the company description. We used three separate keyword filters: “Lung Cancer”, “Breast Cancer”, and “Cancer.” Companies with at least two rounds of funding were selected in order to have enough temporal growth data (*n* = 50 for lung and breast combined; *n* = 484 for general cancer). The second set of the data applied the same techniques as the first except the first filter was removed, so the scope of the companies was global. Finally, we removed irrelevant rounds (“Post-IPO Debt” and “Post-IPO Equity”) from both datasets (*n* = 37 for lung and breast combined; *n* = 333 for general cancer). After pre-processing and cleaning the data, we computed for each company in each dataset both the number of days since the Series A announcement and the total amount of funding raised since Series A funding. We then plotted the total number of days since Series A versus total funding raised since Series A. We computed linear regressions grouping by type of cancer (lung and breast) and cancer overall for both the world and the United States scope.

### Hackathon

Massachusetts Institute of Technology Hacking Medicine (MIT HM) is a student, academic, and community-led organization that pioneered the healthcare hackathon methodology with a focus on system-oriented design thinking to address challenges in healthcare using multidisciplinary teams. In November 2018, MIT HM ran a hackathon in New York City (NY, USA) specifically focused on challenging problems in Lung Cancer. Participants included patients, clinicians, engineers, coders, administrators, lawyers, designers, and public health professionals.

MIT HM hackathons consist of four stages: (1) problem definition, (2) team formation, (3) solution generation and iteration, and (4) presentation. Participants are invited to pitch problems related to lung cancer that they are aware of or have experienced. This can encompass prevention, screening, diagnosis, or treatment. Participants then interact and mingle to form teams organically based on common interests. Diverse, interdisciplinary teams are strongly encouraged. Teams then work to characterize and distill the problem at hand using root cause analyses and stakeholder interviews. Solutions that the teams develop can range from software or hardware technologies to policy or grant proposals. A diverse group of mentors at the event allows teams to iterate their solutions by providing continuous feedback. Teams then present their ideas and solutions to a panel of judges on the final day of the event.

Post-hackathon, a standardized follow-up is conducted by asking participants to complete a codified survey that has been iteratively developed over eight years. This is used to track the progress of teams formed at the event. We assessed the outcome of the hackathon four months after the event by contacting and reviewing progress with event organizers and participants using key informant interviews and surveys. Hackathon participants were informed that data provided through surveys and interviews may be used for research purposes. No informed consent was required as per MIT Committee on the Use of Humans as Experimental Subjects (COUHES), as we report de-identified data. If teams do not complete this survey we assume they are no longer working on their solution. The findings are collated in a large database. Participants retain all intellectual property rights in MIT HM hackathons, to incentivize individuals to participate and contribute.

### Reporting summary

Further information on research design is available in the Nature Research Reporting Summary linked to this article.

## Supplementary information

Supplementary Figures

Reporting Summary

## Data Availability

All data are available from the authors upon reasonable request.

## Code availability

All codes are available from the authors upon reasonable request.
