# Supplementary material for: Grass-roots entrepreneurship complements traditional top-down innovation in lung and breast cancer
Source: NPJ Digit Med. 2022 Jan 21;5:10. doi: 10.1038/s41746-021-00545-x (PMC8782943; doi:10.1038/s41746-021-00545-x)
Supplement: Supplementary file 1 — Supplementary Figures [file 41746_2021_545_MOESM1_ESM.pdf]

Supplementary Figure 1

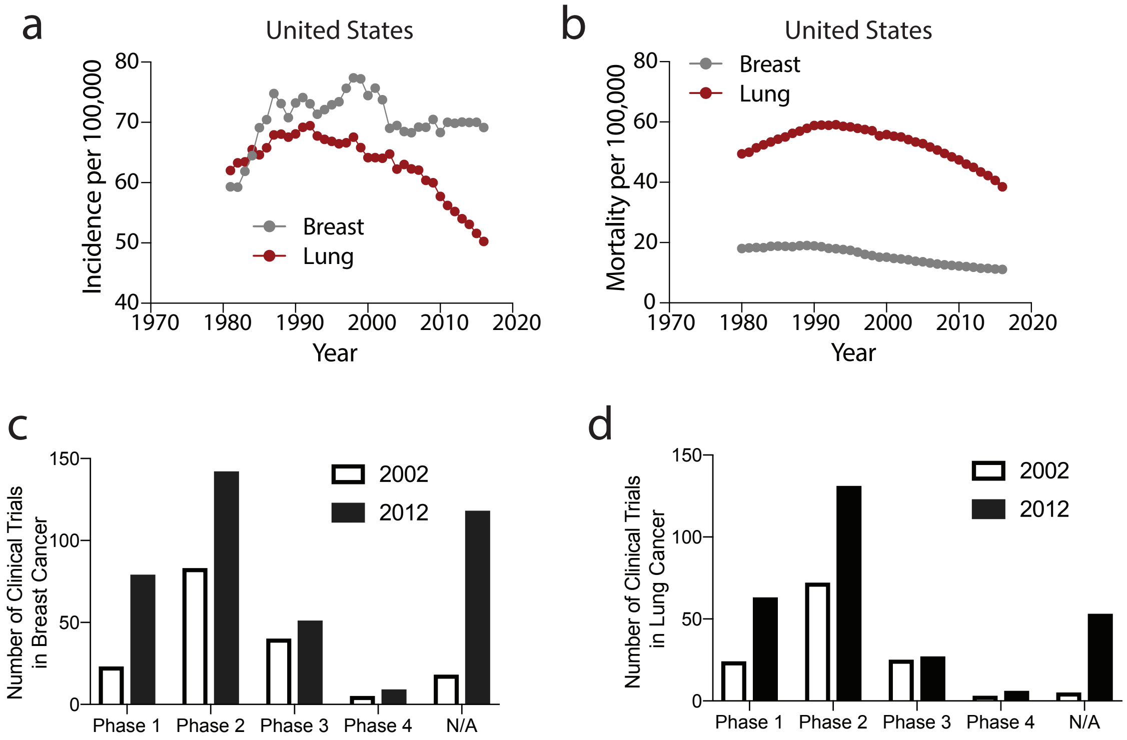

Supplementary Figure 1. (a) Incidence and (b) mortality of breast and lung cancers in the US (1980-present). (c,d) Number of clinical trials in (c) breast and (d) lung cancers over in 2002 and 2012.

Supplementary Figure 2

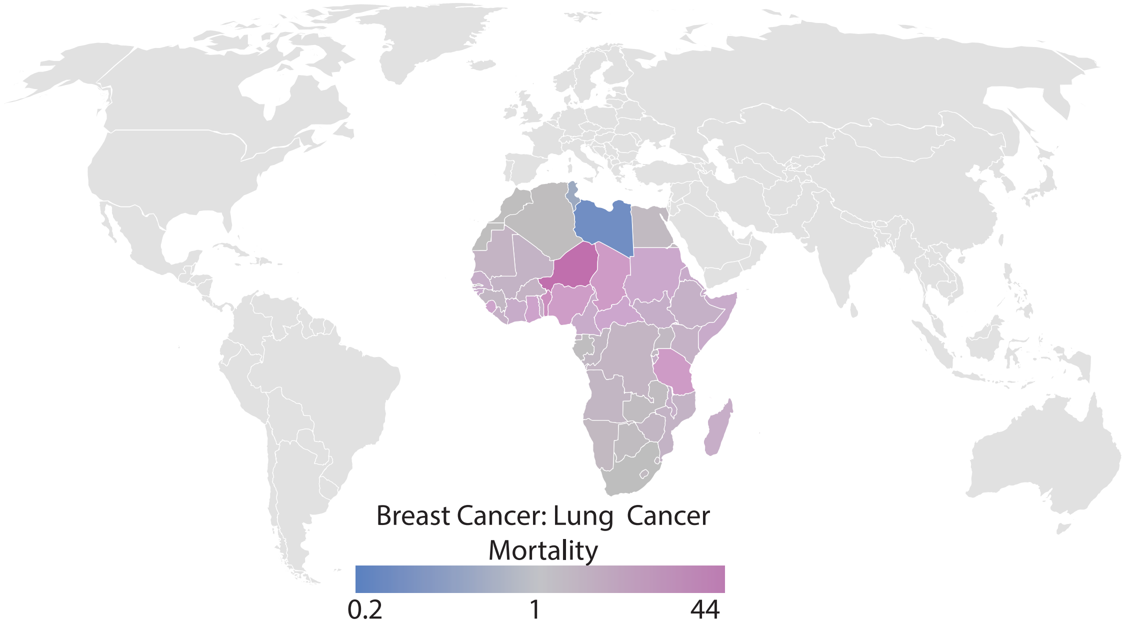

Supplementary Figure 2. Mortality for breast and lung cancer for countries in Africa. Data is shown as the ratio of Breast to Lung. Values higher than 1 (pink) or less than 1 (blue), respectively reflect greater breast cancer, or lung cancer, mortality. Maps generated using Google GeoChart API under Creative Commons Attribution 4.0 License.

Supplementary Figure 3

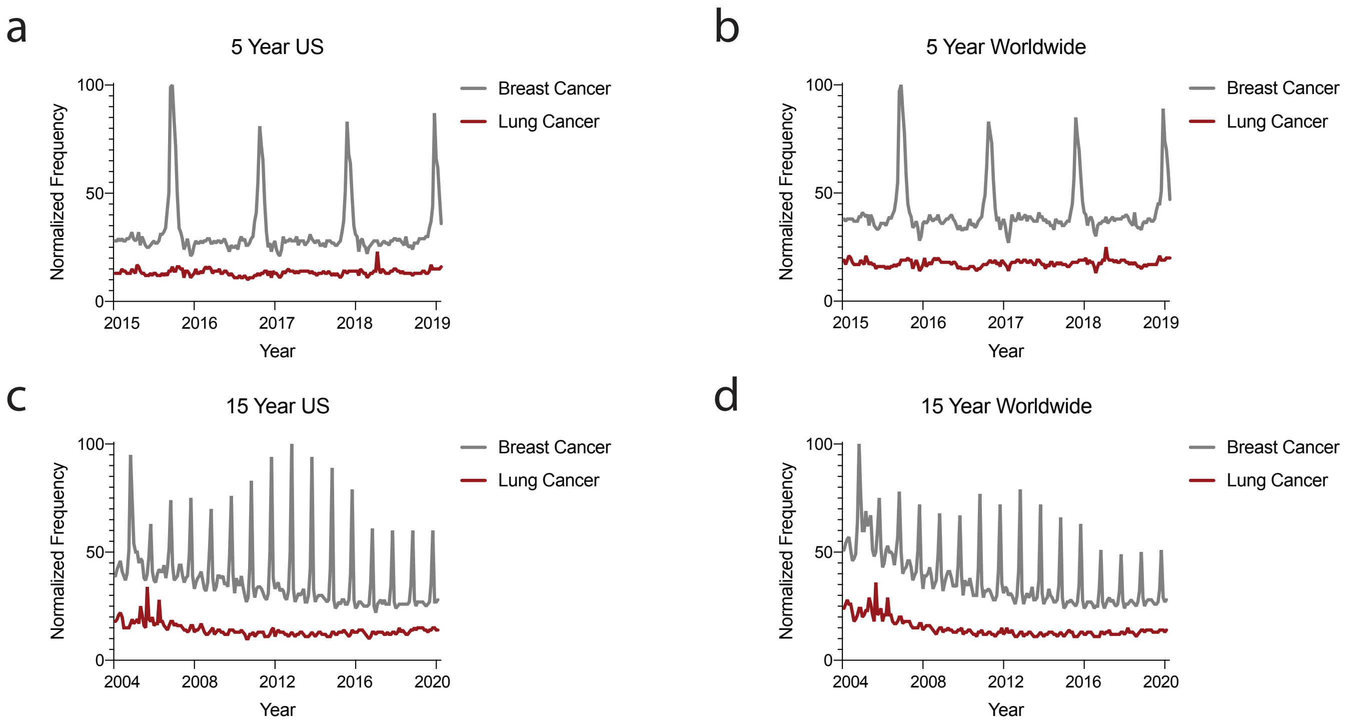

Supplementary Figure 3. Normalized search engine search frequency for “breast cancer” and “lung cancer”. Shown are (a) five-year US, (b) five-year worldwide, (c) fifteen-year US, and (d) fifteen-year worldwide data. Data is normalized to the maximum frequency data point in each graph.

Supplementary Figure 4

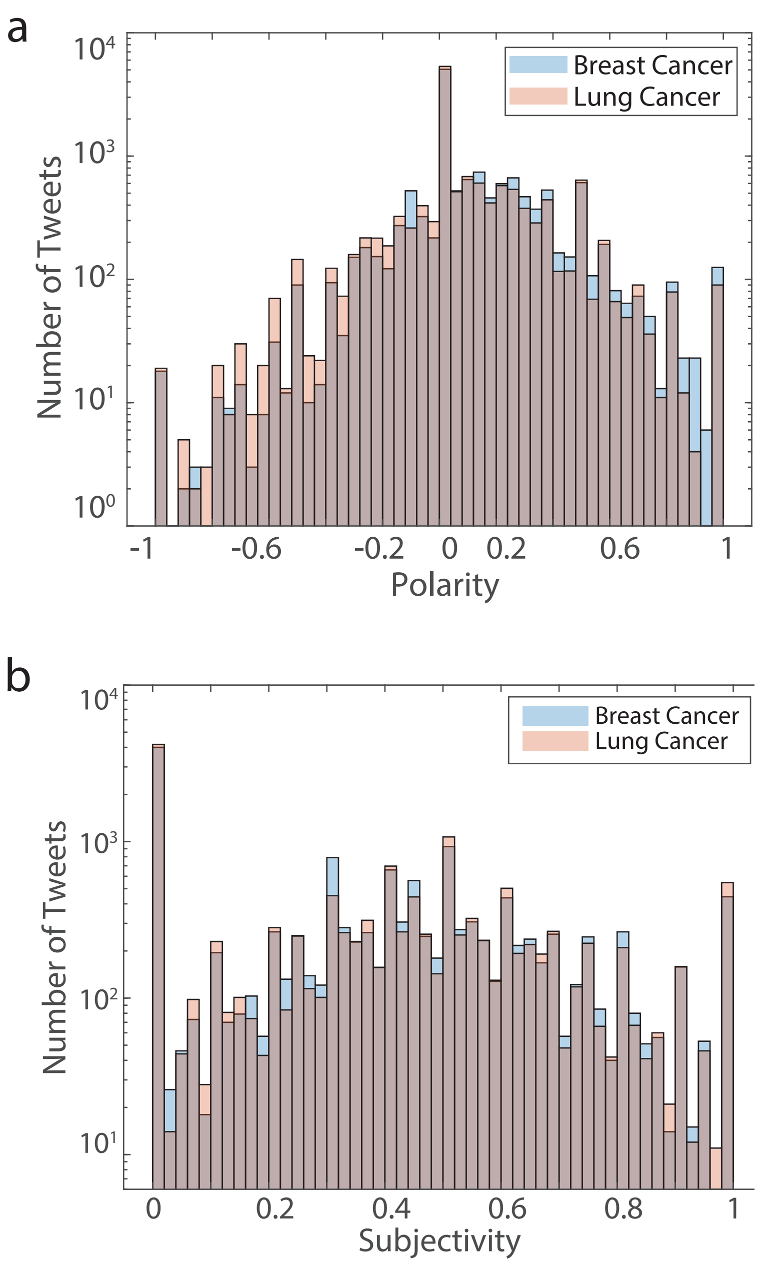

Supplementary Figure 4. Number of breast and lung cancer tweets with various (a) polarity and (b) subjectivity values.

Supplementary Table 1

Team	Innovation Summary
1	A cost effective, easy-to-use device that provides a lung diagnosis within minutes.
2	Machine learning and artificial intelligence software that uses coughs and changes in voice to diagnose respiratory disease and lung cancer.
3	Mobile group therapy to help pregnant women quit and abstain from smoking using real-time detection and social networking.
4	Mobile application that matches pregnant smokers with small personalized communities to quit smoking quickly and prevent relapse.
5	In-home diagnostic technology and navigator for patients to reach out to physician specialists and clinical trial options worldwide.
6	Mobile application that provides a personalized, non-invasive approach to facilitate the early detection of lung cancer by integrating the self-input parameters to quantify pulmonary health.
7	A social platform to empower younger smokers to take control of their own health, reshape social habits and quit smoking, and to influence others to join the movement
8	A nicotine-free alternative to trendy e-cigarettes
9	Online computer-generated radiology reports to evaluate CT scans for Lung Cancer for more diverse groups.
10	Mobile CT screening center to 20 underserved urban centers in the US.
11	An application-based rewards program to improve lung cancer prognosis
12	An device to detect exact smoke inhalation tethered to an app with social media and data analytics functionality to encourage smoking cessation.
13	Noninvasive, cost effective device using breath analyzer bag to diagnose lung cancer.

Supplementary Table 1. List of innovations generated at 2018 MIT HM New York City Grand Hack focused on lung cancer. Innovation summaries are self-reported by teams.
